# Role of C-Terminal Phosphorylation of Lamin A in DNA Damage and Cellular Senescence

**DOI:** 10.3390/cells12040639

**Published:** 2023-02-16

**Authors:** Ying Ao, Zhuping Wu, Zhiwei Liao, Juncong Lan, Jie Zhang, Pengfei Sun, Baohua Liu, Zimei Wang

**Affiliations:** 1 Guangdong Key Laboratory for Genome Stability & Human Disease Prevention, Carson International Cancer Center, Department of Biochemistry & Molecular Biology, School of Basic Medical Sciences, Shenzhen University Medical School, Shenzhen 518055, China; 2 Shenzhen Key Laboratory for Systemic Aging and Intervention, National Engineering Research Center for Biotechnology (Shenzhen), Shenzhen University, Shenzhen 518055, China; 3 Shenzhen University-Friedrich Schiller Universität Jena Joint PhD Program, Friedrich Schiller Universität, 07743 Jena, Germany

**Keywords:** lamin A, phosphorylation, DNA damage, cellular senescence, premature aging

## Abstract

The nuclear matrix protein lamin A is a multifunctional protein with roles in DNA replication and repair, gene activation, transcriptional regulation, and maintenance of higher-order chromatin structure. Phosphorylation is the main determinant of lamin A mobility in the nucleus and nuclear membrane dissolution during mitosis. However, little is known about the regulation of lamin A phosphorylation during interphase. Interestingly, C-terminal lamin A mutations trigger cellular senescence. Recently, we showed that the C-terminal region of lamin A interacts with casein kinase II (CK2). In the present study, we have expanded on our previous research to further investigate lamin A phosphorylation and elucidate the mechanisms underlying the effect of C-terminal mutations on cellular senescence. Our results indicate that glycogen synthase kinase 3β (GSK3β) and CK2 jointly mediate the phosphorylation of lamin A at C-terminal Ser628 and Ser636 residues. Furthermore, a loss of phosphorylation at either of these two sites affects the nuclear distribution of lamin A, leading to an impaired DNA damage response as well as cellular senescence. Thus, phosphorylation at C-terminal sites in lamin A appears to be important for maintaining genomic stability and preventing cellular senescence. These findings provide insight into how loss of the C-terminal region of lamin A may induce premature aging. Furthermore, enhancement of GSK3β and CK2 activity may represent a possible therapeutic approach for the treatment of aging-related diseases.

## 1. Introduction

The nuclear lamina has roles in nuclear integrity, mitosis, mechano-sensation, DNA replication, signaling, regulation of transcription, and genome organization [1,2]. Lamin A, a member of the cytoskeletal intermediate filament class of proteins, is mainly found in the nuclear lamina, a structure located below the inner layer of the nuclear membrane. Mature lamin A protein consists of 646 amino acids and its molecular structure includes an N-terminal spherical domain (head), an intermediate helical rod-like domain, and a C-terminal globular domain (tail) [3]. Together with lamin B and lamin C, head-to-tail dimers of lamin A form a fibrin sheet or network that maintains the shape of the nucleus and the nuclear membrane [4]. Lamin A is important for stabilizing and anchoring perinuclear heterochromatin [5] and plays roles in development, cell differentiation, aging, and disease. Accordingly, mutations of lamin A can cause several diseases such as progeria, metabolic disorders, and muscular dystrophy [6]. Hutchinson–Gilford progeria syndrome, a fatal autosomal dominant human disorder characterized by premature aging features, is predominantly caused by a de novo G608G point mutation in the LMNA gene, which encodes A-type lamin proteins [7]. The G608G mutation activates a cryptic splice donor in exon 11 of the LMNA gene, resulting in a 50-amino acid internal deletion proximal to the C-terminus of the lamin A protein and the production of an aberrant prelamin A called progerin [7,8]. In normal cells, progerin accumulation can inhibit autophagy, aggravate DNA damage, and accelerate the aging process [9]. The hallmarks of cellular senescence include DNA damage response (DDR), CDK inhibitors and cell cycle arrest, secretory phenotype, apoptosis resistance, metabolism, and endoplasmic reticulum stress [10]. Normal aging and progeria syndrome share many characteristics, such as an accumulation of DNA damage, epigenetic changes, and a depletion of stem cells etc. [11]. Indeed, there is increasing evidence to show that research on progeria may have significance for the understanding of normal aging mechanisms.

Studies have shown that the depolymerization and nuclear localization of lamin A are closely related to its phosphorylation level [12,13,14,15]. Several serine and threonine residues within specific regions of the lamin A protein can undergo phosphorylation; these include N-terminal Ser12, Thr14, Ser17, and Ser22 sites, as well as Ser385, Ser387, Ser390, Ser392, Ser403, Ser404, and S423 sites located within the middle helical rod-shaped domain, and further sites at the C-terminal domain [16,17]. In particular, phosphorylation of Ser22 and Ser392, known as the mitotic sites, is involved in mediating lamin A depolymerization and dissolution, which mark the initiation of cell division (G2/M) [18,19]. In addition, phosphorylation of the C-terminal Ser628 site causes the rapid migration of lamin A from the nuclear envelope to the nucleoplasm and has synergistic effects on lamin A migration when combined with the phosphorylation of Ser22 and Ser392 [19]. Despite these reports, the role of phosphorylation in lamin A function has not been fully elucidated. Interestingly, several phosphorylation sites in the C-terminal region of lamin A overlap with the 50 amino acids that are absent in progerin [19]. Although previous studies have focused more on the gain of function of progerin, the possibility that important phosphorylation sites located within the 50 missing amino acids may play a role in cellular senescence or premature aging is worthy of further exploration.

Through binding to a variety of nuclear proteins, lamin A acts as a signal integration platform. As such, phosphorylation of lamin A may have a direct impact on the binding sites of several proteins, affecting the transport of a series of important proteins in the nucleus and leading to changes in downstream signaling pathways. Studies have shown that phosphokinases that catalyze phosphorylation at specific lamin A sites are likely to directly participate in the regulation of activities such as the cell cycle and cellular senescence via the lamin A molecular platform. For example, the phosphokinase Cdk1 catalyzes the phosphorylation of N-terminal Ser22 and Ser392 sites in lamin A/C—an early event in cell mitosis [13]. Additionally, PKC and Akt are known to phosphorylate lamin A at specific sites such as Ser403, Ser404, Ser406, and Ser407 [20]. However, it is unclear which phosphokinases are responsible for the phosphorylation of sites within the C-terminal domain of lamin A. Our previous research found that the protein kinase CK2 binds the nuclear lamina, and its activity affects cellular senescence and life span at the organismal level. CK2α knockdown accelerates senescence, and CK2α over-expression attenuates senescence and provides a novel function for nuclear lamin A, i.e., buffering CK2 kinase activity, highlighting an essential role for CK2 in the regulation of senescence and organismal aging [21]. Therefore, in this study, we investigated the phosphorylation of lamin A at the C-terminus, aiming to identify the phosphokinases involved as well as the effects of phosphorylation on lamin A function. We hypothesize that the C-terminal phosphorylation of wild type lamin A protects against cell senescence and that disabling the phosphorylation sites negates this protection.

## 2. Materials and Methods

### 2.1. Mouse Mutants and MEF Culture

The *LMNA^Pro/+^* allele (Lmna^G609G^ mutation flanked by two loxP sites) was generated by Cyagen Biosciences Inc., China. The G609G (GGC to GGT) mutation was introduced into exon 11 in the 3′homology arm. C57BL/6 embryonic stem cells were used for gene targeting [22]. *LMNA^+/−^* intercrossed mice (C57BL background) were obtained from Z. Zhongjun (Hong Kong University).

HEK 293 cell lines (CRL-1573) were purchased from ATCC. *LMNA^Pro/Pro^* and *LMNA*^−/−^ MEFs were isolated separately from 13.5-day-old embryos produced by *LMNA^Pro/+^* and *LMNA^+/−^* intercrossed mice, respectively (on a C57BL background). Littermate-matched *LMNA^Pro/Pro^, LMNA*^+/+^, and *LMNA*^−/−^ MEFs were cultured in Dulbecco’s Modified Eagle Medium (DMEM) containing 10% fetal bovine serum (FBS). Cells were cultured at 37 °C in 5% CO_2_ and atmospheric oxygen conditions.

### 2.2. Plasmid Construction

Lamin A and progerin sequences were amplified and cloned into p3xFLAG-CMV or pGEX-4T-3 vectors. PCR-based deletion of the FLAG-Lamin A plasmid template was used to generate truncated forms of lamin A protein. Lamin A and point-mutated lamin A sequences were cloned into the lentiviral vector pLVX-mCherry-C1 by PCR. Transient transfections of these plasmids were performed using Lipofectamine 3000 (Invitrogen, Carlsbad, CA, USA) according to the manufacturer’s protocol. Primer sequences for the mutation of lamin A were as follows:

LMNA-S625A-F: 5′-CCACACTGCGGTAGGCGCGAGTGACCGTGA-3′
LMNA-S625A-R: 5′-TCACGGTCACTCGCGCCTACCGCAGTGTGG-3′
LMNA-S628A-F: 5′-ACTGCCCCCCACAGCGCGGTAGCTGCGA-3
LMNA-S628A-R: 5′-TCGCAGCTACCGCGCTGTGGGGGGCAGT-3′
LMNA-S632A-F: 5′-GCTGCCACCCCCAGCGCCCCCCACACTG-3
LMNA-S632A-R: 5′-CAGTGTGGGGGGCGCTGGGGGTGGCAGC-3′
LMNA-S636A-F: 5′-GCAGTGGGGGTGGCGCCTTCGGGGACAATC-3′
LMNA-S636A-R: 5′-GATTGTCCCCGAAGGCGCCACCCCCACTGC-3′
LMNA-S652A-F: 5′-CTGGGTTCGGGGGGCGGAGTTGCCCAGG-3′
LMNA-S652A-R: 5′-CCTGGGCAACTCCGCCCCCCGAACCCAG-3′

### 2.3. Antibodies

Anti-lamin A/C (sc-20681), anti-p16^INK4a^ (sc-1661), and anti- p21^WAF1/CIP1^ (sc-6246) antibodies were purchased from Santa Cruz Biotechnology (Santa Cruz, CA, USA); anti-γH2AX (05-636) was sourced from EMD Millipore (Boston, MA, USA); and anti-KAP-1 (ab10484), anti-p-KAP-1 Ser824 (ab70369), anti-p-S/T (ab17464), anti-GSK3β (ab93926), and anti-GST (ab108524) antibodies were obtained from Abcam (Cambridge, UK). Mouse anti-HA and anti-FLAG antibodies were obtained from Sigma-Aldrich, while mouse anti-actin and anti-GAPDH antibodies were obtained from Beyotime (Shanghai, China). Anti-pS628 and anti-pS632 lamin A polyclonal antibodies were generated by GenScript (Nanjing, China) using a specific phosphorylated peptide (Supplementary Figures S4 and S5).

### 2.4. Western Blotting

Cell extracts for Western blotting were prepared using modified RIPA buffer (0.2 M NaCl, 0.1 M Tris-HCl [pH 7.5], 1mM EDTA, 1 mM DTT, phosphatase inhibitors, and protease inhibitors), boiled in sodium dodecyl sulfate (SDS) sample loading buffer, and resolved on SDS polyacrylamide gels before being transferred to PVDF membranes (Millipore). The membranes were blocked in 3% BSA in Tris-buffered saline with Tween 20 (TBST; 20 mM Tris–HCl [pH 7.6], 150 mM NaCl, 0.05% Tween 20) for 1 h at room temperature and incubated with primary antibodies (the antibody concentration was 1 µg/mL) overnight at 4 °C. The membranes were then probed with the respective HRP-linked secondary antibodies for 1 h at room temperature. Immunoreactive products were visualized using an enhanced chemiluminescence kit (Pierce 34095) and a Bio-Rad imaging system (ChemiDoc XRS +, 1708265).

### 2.5. Immunoprecipitation

For immunoprecipitation, cells exposed to the indicated treatments were harvested in lysis buffer (20 mM Tris-HCl [pH 7.5], 250 mM NaCl, 0.1 mM EDTA, 10% glycerol, and 0.1% NP-40) plus phosphatase inhibitors and protease inhibitors (Roche Complete). Cell lysates were incubated with 1 µg of the respective antibodies or control IgGs at 4 °C overnight. Then, bead-bound immunoprecipitates were washed with lysis buffer, after which the beads were boiled in SDS sample loading buffer and analyzed by Western blotting.

### 2.6. Protein Purification and Pull-Down Assays

GST-fusion proteins (bait and prey) were expressed in BL21 bacterial cells (induced by isopropyl beta-D-thiogalactopyranoside) and purified using glutathione-Sepharose 4B beads (GE Healthcare, Fairfield, CT, USA), with two washes in TEN buffer (20 mM Tris-HCl [pH 7.4], 0.1 mM EDTA, and 100 mM NaCl). For GST pull-downs, 1 µg GST, GST-lamin A, or GST-lamin A mutant protein was immobilized on Glutathione Sepharose 4B beads (GE) and incubated with 6× His-tagged GSK3β (purified from bacterial culture) at 4 °C overnight in GST-binding buffer (50 mM Tris-HCl, pH 7.5, 0.2 mM EDTA, 150 mM NaCl, and 0.1% NP-40). The beads were washed four times with GST-binding buffer and then analyzed by Western blotting with the indicated antibodies.

### 2.7. In Vitro Kinase Assay

Kinase reactions were initiated by incubating purified CK2α or GSK3β with GST-lamin A C-terminal protein and mutants in kinase buffer (kinase buffer for CK2α: 20 mM Tris-HCl, 0.5 mM DDT, 20 mM MgCl_2_, and 0.2 mM ATP; kinase buffer for GSK3β: 25 mM Tris-HCl, 2 mM β-mercaptoethanol, 1 mM MgCl_2_, 0.5 mM ATP, and 5% glycerol). Each reaction required 6 µg substrate protein and 60 ng kinase in a total volume of 30 µL (supplemented with ddH_2_O); the samples were then mixed well and incubated at 30 °C for 2 h. The reactions were blocked by the addition of SDS loading buffer and the samples were analyzed by Western blotting; antibodies specific for phosphorylated lamin A were used to probe the membranes.

### 2.8. Senescence-Associated β-Gal Assay

Senescence-associated β-galactosidase activity was assessed using a cellular senescence assay kit (CST, #9860) according to manufacturer’s instructions. Briefly, cells were seeded in 6-well plates, fixed at room temperature for 15 min with 2 mL of 1× fixing solution, washed three times with 1× PBS, and stained with 1 mL of freshly prepared 1× β-galactosidase detection solution at 37 °C for 16 h in the dark. The cells were then washed twice with 1× PBS, overlaid with 70% glycerol/PBS, and photographed. The number of stained cells among more than 300 randomly chosen cells were counted. The data were analyzed using the two-tailed Student’s *t*-test.

### 2.9. Statistical Analyses

At least three replicates were carried out for all experiments. Data are presented as means ± SD or means ± SEM (as indicated). Statistical analyses were conducted using the two-tailed Student’s *t*-test to show differences between two groups, and the following *p* values were considered statistically significant: * *p* < 0.05, ** *p* < 0.01, and *** *p* < 0.001. Statistical analyses were performed using Graphpad Prism software v.5.03.

## 3. Results

### 3.1. Lower Level of Phosphorylation in Progerin Compared with Lamin A

**Figure 1:**
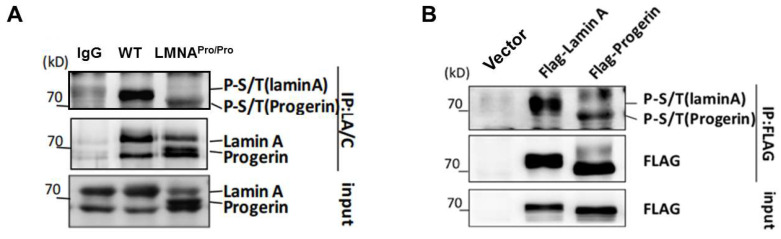
Decreased phosphorylation of progerin compared with lamin A. (**A**) Western blots showing the phosphorylation levels of wild type lamin A and LMNA^pro/pro^ (progerin) in MEF cells, detected by a universal serine/threonine antibody. (**B**) FLAG-lamin A and FLAG-progerin were expressed in HEK293 cells and immunoprecipitated using an anti-FLAG antibody; The universal serine/threonine antibody was used to detect the phosphorylation levels of progerin and lamin A.

Since lamin A and progerin have different functions in DNA damage repair, regulation of gene activation, and transcription, we tried to determine whether these differences were related to their phosphorylation level. Therefore, we investigated the phosphorylation levels of full-length lamin A expressed in wild type MEFs and progerin expressed in MEFs obtained from LMNA^pro/pro^ mice (Figure S1A–C). Immunoprecipitation was performed using antibodies against lamin A/C, and the phosphorylation levels of lamin A/C were determined using universal serine–threonine antibodies. The results showed that progerin exhibited a significantly lower level of phosphorylation than lamin A (Figure 1A). Furthermore, transfection of FLAG-lamin A or FLAG-progerin overexpression vectors in 293T cells followed by in vitro immunoprecipitation confirmed the reduced phosphorylation of progerin (Figure 1B). These results suggested the presence of phosphorylation sites within the 50 amino acids missing from progerin.

**Figure 2:**
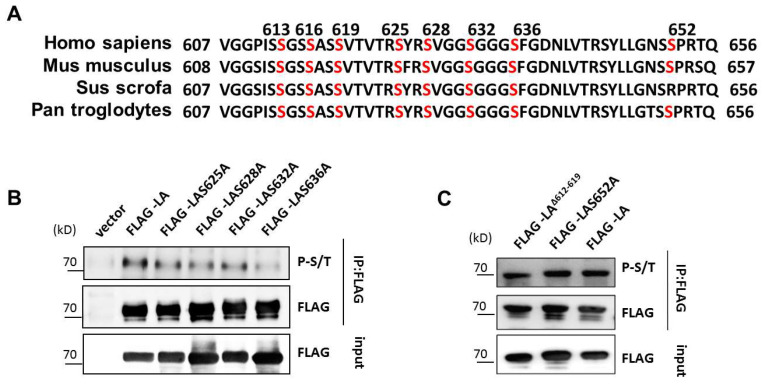
Influence of Ser628 and Ser636 on the phosphorylation level of lamin A. (**A**) Analysis of potential phosphorylation sites among the 50 C-terminal amino acids of the lamin A protein. (**B**,**C**) FLAG-lamin A and its mutants were expressed in HEK293 cells and immunoprecipitated using an anti-FLAG antibody; Western blot analysis of the phosphorylation levels of wild type lamin A and lamin A-containing mutated phosphorylation sites using antibodies specific for serine/threonine phosphorylation.

To identify potential phosphorylation sites within this 50-amino acid region, we analyzed the lamin A sequence in different species and found the C-terminal sequence to be relatively conserved in mammals (Figure 2A). Then, we used the online tools DISPHOS (http://www.dabi.temple.edu/disphos/, accessed on 10 February 2017), NetPhos3.1 (http://www.cbs.dtu.dk/services/NetPhos, accessed on 10 February 2017), and PhosphoSitePlus (https://www.phosphosite.org/homeAction.action, accessed on 10 February 2017) to identify phosphorylation sites within the 50-amino acid region (Figure S2A–C). The results indicated that Ser613, Ser616, Ser619, Ser625, Ser628, Ser632, Ser636, and Ser652 could be potential phosphorylation sites. Therefore, we went on to construct lamin A expression constructs with serine-to-alanine mutations at these sites. Immunoprecipitation experiments were conducted to isolate lamin A and mutant proteins from transfected 293T cells, and the phosphorylation of lamin A was detected using a universal phosphorylation antibody. The results showed markedly reduced phosphorylation of lamin A with mutations in either Ser628 or Ser636 sites (Figure 2B,C), in line with the NetPhos3.1 prediction. Ser628 has been reported to be a high-turnover site, while mutations of Ser628 in conjunction with Ser22 and Ser392 mutations in lamin A are known to affect nuclear and extranuclear localization [19]. Therefore, subsequent experiments focus on phosphorylation at Ser628.

### 3.2. Interaction between Lamin A and GSK3β

Based on an analysis of the results obtained from five phosphorylation prediction tools, we set out to identify the kinases responsible for the phosphorylation of lamin A at Ser628 (Figure S3A). While GSK3β and CK1 appeared frequently in various predictions (Figure S3A), only GSK3β possessed the consensus motif required for targeting the sequences surrounding the Ser628 site (Figure S3B). Therefore, we speculated that GSK3β may be involved in regulating the phosphorylation of the Ser628 site of lamin A.

**Figure 3:**
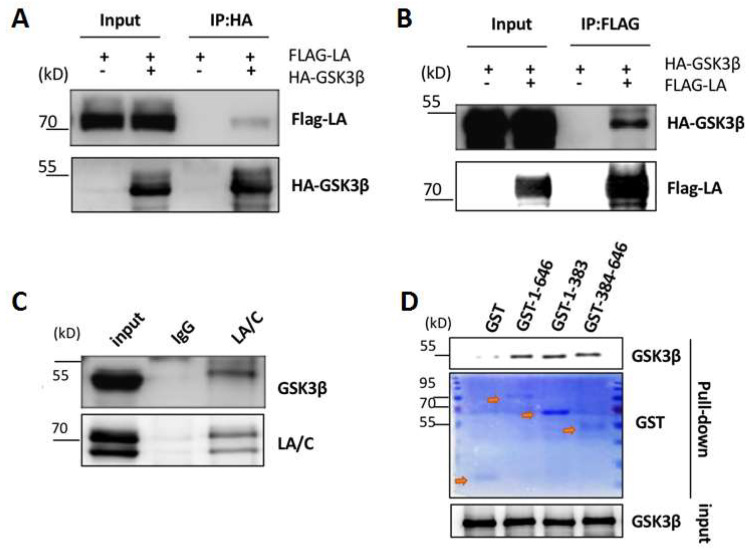
Direct interaction between GSK3β and lamin A. (**A**,**B**) Representative co-immunoprecipitation (co-IP) and Western blots for HEK293 cells transfected with HA-GSK3β and FLAG-lamin A, showing proteins immunoprecipitated with anti-HA (**A**) or anti-FLAG (**B**) antibodies. (**C**) Representative co-IP and Western blots showing the interaction between GSK3β and lamin A in MEF cells in vivo; GSK3β was pulled down by anti-lamin A/C antibodies. (**D**) GST fusions of full-length and truncated lamin A were subjected to His-GSK3β pull down. Western blotting was performed to detect GSK3β protein levels and Coomassie brilliant blue (CBB) staining was performed to detect GST, GST-lamin A, and GST-lamin A mutant-tagged proteins; red arrows indicate the corresponding protein bands.

To determine whether these two proteins were able to interact, we expressed FLAG-lamin A and HA-GSK3β proteins in 293T cells and analyzed their interaction by co-immunoprecipitation assays. The results showed that lamin A was detected in anti-HA-tag immunoprecipitates, while, conversely, GSK3β was detected in samples pulled down with anti-FLAG antibody (Figure 3A,B), indicating an interaction between the two proteins. To confirm this result, whole-cell lysates prepared from wild type MEFs were incubated with lamin A/C antibodies or IgG antibodies (as controls). Immunoprecipitation revealed that GSK3β protein was detected in the presence of lamin A (Figure 3C), indicating that GSK3β and lamin A were able to interact in vivo. Furthermore, pull-down experiments using purified GST fusion proteins of full-length lamin A (amino acids 1-646) or truncated lamin A (N-terminal [amino acids 1–383] or C-terminal [amino acids 384–646] domains) in the presence of His-GSK3β showed a direct interaction between GSK3β and the C-terminal region of lamin A (Figure 3D).

### 3.3. Role of GSK3β in the Phosphorylation of Lamin A at Ser628

**Figure 4:**
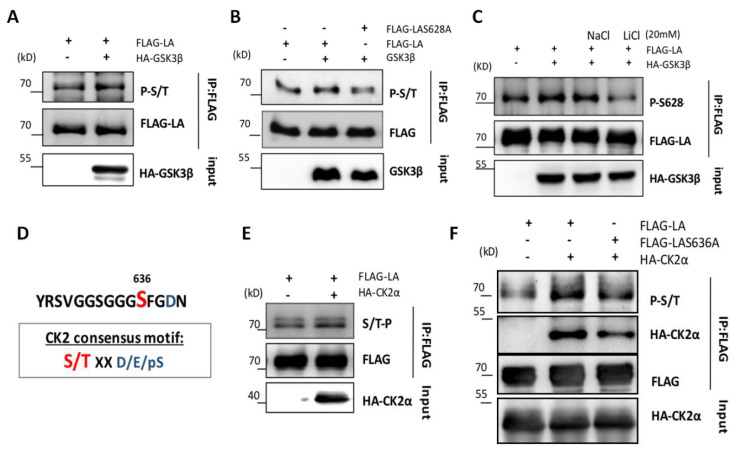
GSK3β-induced phosphorylation of lamin A at Ser628 and CK2α-induced phosphorylation of lamin A at Ser636. (**A**) Immunoprecipitation of FLAG-lamin A and HA-GSK3β expressed in HEK293 cells was performed using FLAG antibodies; phosphorylation levels were detected using a serine/threonine phosphorylation-specific antibody. (**B**) Immunoprecipitation of mutant lamin A (FLAG-LAS628A) and GSK3β expressed in HEK293 cells was performed using FLAG antibodies; phosphorylation levels were detected using a serine/threonine phosphorylation-specific antibody. (**C**) An antibody specific to phosphorylated Ser628 was used to detect lamin A phosphorylation. (**D**) The CK2α consensus motif conforms to the Ser636 site. (**E**) The level of lamin A phosphorylation was detected following FLAG antibody immunoprecipitation from 293T cells co-expressing HA-CK2α and FLAG-lamin A. (**F**) The level of lamin A phosphorylation was detected following FLAG antibody co-immunoprecipitation from 293T cells co-expressing HA-CK2α and mutant lamin A. All transfections were expressed in HEK293 cells.

To explore whether GSK3β was able to catalyze the phosphorylation of Ser628, we co-transfected 293T cells with HA-GSK3β and FLAG-lamin A or FLAG-lamin A- S628A plasmids. Immunoprecipitation revealed reduced phosphorylation of lamin A-S628A compared with wild type lamin A (Figure 4A,B). This finding implied that GSK3β was phosphorylating lamin A at the Ser628 site.

To further confirm phosphorylation of Ser628 by GSK3β, we generated a polyclonal antibody specific to Ser628-phosphorylated lamin A (Figure S4A,B). Then, 293T cells were transfected with wild type lamin A and GSK3β constructs and incubated with the GSK3β inhibitor lithium chloride (20 mM) or ddH_2_O as a negative control for 4 h and IP of lamin A from cell lysates using an anti-FLAG antibody. The phosphorylation of lamin A was detected using the Ser628-specific phosphorylation antibody. The level of phosphorylation at Ser628 was found to be markedly decreased in the presence of lithium chloride but not ddH_2_O (Figure 4C), confirming our previous findings.

In addition, to verify the role of GSK3β in the phosphorylation of lamin A Ser628, we conducted in vitro kinase assays using Ac-SYRS(P)VGGSGGC-NH2 peptides. However, when the peptides were incubated together in vitro, phosphorylation at Ser628 was not apparent in the presence of GSK3β alone (Figure S4C). As GSK3β kinase substrates have been shown to require an initial phosphorylation of serine or threonine present at +4 residues from the carboxyl side, we speculated that Ser636 may play a priming role in Ser628 phosphorylation. Ser632 and Ser636 activation (mutation of the S residues to D) and inactivation peptides (mutation of the S residues to A) and wild type controls (LA 624-640) were synthesized to verify this possibility. Increased phosphorylation of Ser628 by GSK3β was evident in the presence of Ser636 and Ser632 phosphorylation-activated peptides, while Ser628 phosphorylation signals were weak or absent in the presence of Ser636- and Ser632-inactivated peptides. These findings imply that activation of the carboxyl-terminal site Ser636 of lamin A may promote GSK3β-induced phosphorylation of the Ser628 site (Figure S4D).

According to the online phosphorylation prediction tools used previously, we found that the lamin A Ser636 site was likely to be a substrate for phosphorylation by CK2α. CK2α catalyzes the phosphorylation of substrates with negatively charged amino acids such as aspartic acid (D), glutamic acid (E), or phosphoserine (pS) located at +1 or +3 residues on the carboxyl side (Figure 4D). Previous research by our group demonstrated that CK2α binds directly to the C-terminus of lamin A [21]. Here, transfection and immunoprecipitation experiments carried out in 293T cells showed that the phosphorylation of wild type lamin A was increased in cells overexpressing CK2α (detected with a universal serine/threonine antibody; Figure 4E). However, phosphorylation of the Ser636A-mutated form of lamin A was reduced compared with wild type lamin A, and its interaction with CK2α was markedly weakened (Figure 4F).

### 3.4. CK2-Induced Phosphorylation of S636 Triggers GSK3β-Induced Phosphorylation of Ser628

**Figure 5:**
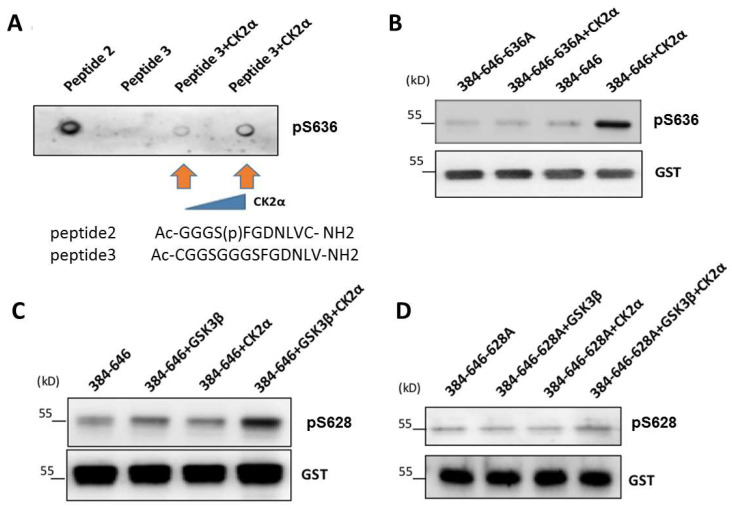
GSK3β-induced phosphorylation of lamin A at Ser628 triggered by CK2-induced phosphorylation at Ser636. (**A**) In vitro kinase assays carried out using phosphorylated antibodies of Ser636 indicated that CK2 induced phosphorylation at Ser636. (**B**) In vitro kinase assays using phosphorylated antibodies of Ser636 to detect substrates of GST fusion to the lamin A C-terminal domain demonstrated that GSK3β induced phosphorylation at Ser628. (**C**,**D**) In vitro kinase assays using phosphorylated antibodies of Ser628 to detect the substrates of GST fused to the lamin A C-terminal domain with or without mutation of Ser628. Ser636 phosphorylation by CK2 was triggered by phosphorylation of Ser628 by GSK3β. In all assays, phosphorylation levels were detected using phosphorylated antibody of Ser628 or Ser636.

To further examine phosphorylation of the lamin A Ser636 site by CK2α, we generated an antibody against Ser636-phosphorylated lamin A and verified its specificity (Figure S5A,B). In vitro kinase assays using Ac-GGGS(P)FGDNLVC-NH2 showed that peptide phosphorylation at Ser636 was increased after the addition of recombinant CK2α. In addition, in vitro kinase assays performed using GST tag-purified lamin A C-terminal protein and mutants showed that phosphorylation at Ser636 of lamin A was increased after the addition of CK2α (Figure 5A,B).

The above results demonstrated phosphorylation of lamin A at the Ser636 site by CK2α both in vivo and in vitro. We then investigated the role of Ser636 in activating phosphorylation at Ser628 through a subsequent series of in vitro kinase experiments using GST tag-purified lamin A C-terminal protein and mutants. In the presence of either CK2α or GSK3β, phosphorylation at Ser628 did not change. In contrast, when both CK2α and GSK3β were added, phosphorylation at Ser628 was markedly increased (Figure 5C,D). These results suggested that the phosphorylation of lamin A at Ser636 by CK2α triggered the activation and phosphorylation of Ser628. Thus, Ser636 may act as a priming site, with an important role in the C-terminal phosphorylation of lamin A.

### 3.5. Role of Loss of Phosphorylation of Lamin A at Ser628 and Ser636 in Cellular Senescence

**Figure 6:**
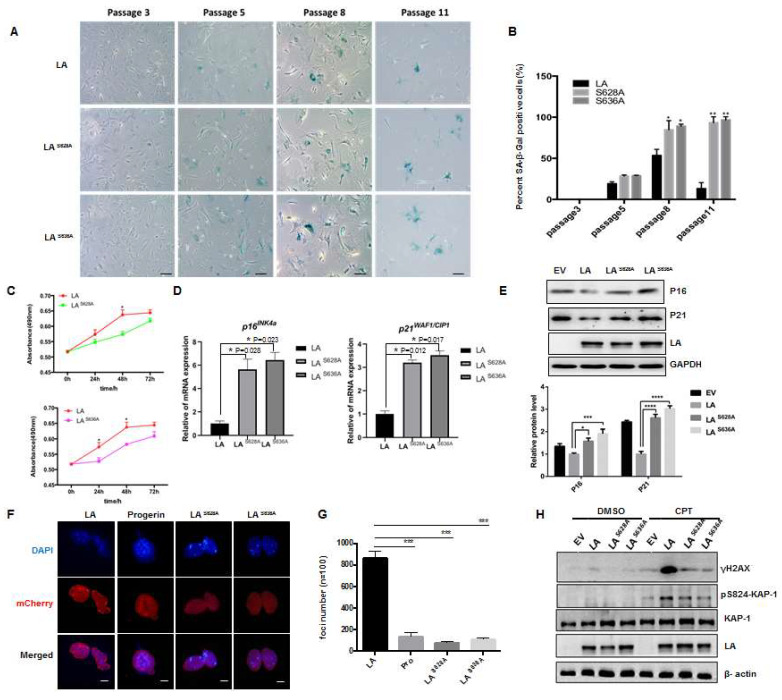
Role of lamin A phosphorylation at Ser628 and Ser636 in cellular senescence. (**A**) Representative images of SA-β-gal activity showing blue-stained senescent cells in Ser628/Ser636-mutant lamin A-expressing MEFs and wild type controls (scale bar, 100 μM). (**B**) Quantification of SA-β-Gal-positive staining (**A**) from six views randomly captured for each group. Data represent means ± SEM. * *p* < 0.05, ** *p* < 0.01. (**C**) Graph showing differences in the proliferative ability of MEF cells expressing mutant and wild type lamin A, determined by MTS assay. (**D**) Quantitative RT-PCR analysis of mRNA levels of the indicated *p16^INK4a^* and *p21^WAF1/CIP1^* genes in cells expressing mutant and wild type lamin A MEFs. Data represent means ± SEM. * *p* < 0.05. (**E**) Western blot assay showing the levels of senescence marker proteins in cells expressing mutant and wild type lamin A. EV is empty vector plvx-mCherry-C1. Quantification of P16 and P21 protein expression are shown below. Quantification was performed by Image J; data were presented as means ± S.D. The experiments were repeated three times with consistent results. *** *p* < 0.001, **** *p* < 0.0001. (**F**) Representative immunofluorescence confocal microscopy images of MEF cells expressing Ser628/Ser636-mutant lamin A or wild type lamin A and progerin (scale bar, 10 µm). (**G**) Quantification of lamin A immunofoci from ten randomly selected views for each group. At least 100 cells were counted; the line chart shows the results of three independent experiments (means ± SEM). *** *p* < 0.001. (**H**) Representative immunoblots showing levels of γH2AX and p824-KAP1 in MEF cells expressing Ser628/Ser636-mutant lamin A or wild type lamin A after treatment with 4 μM camptothecin (CPT) for 1 h to induce DNA damage (or vehicle DMSO as a negative control); EV is empty vector plvx-mCherry-C1. At least three independent experiments were performed.

To verify the function of lamin A-specific phosphorylation, we obtained primary MEFs from the LMNA^−/−^ mouse model and used them to generate cell lines expressing wild type lamin A or mutant proteins with phosphorylation site-specific mutations tagged with mCherry (Figure S6A). LMNA^−/−^ MEFs were transfected with lentiviral recombinant plasmids containing the target genes along with the viral packaging plasmids psPAX2/pMD2.G; the cells were screened with puromycin (1 μg/mL), and the levels of lamin A protein and mutant proteins were determined by fluorescence and Western blot experiments. The results showed that cell lines expressing lamin A or point-mutated lamin A (LA^S628A^ and LA^S636A^) were successfully constructed (Figure S6B,C). With the aid of β-gal staining assays, the degree of senescence in wild type and mutant lamin A cells was then determined at different passages. The cells began to exhibit senescence after passage 5, with the senescence of mutant cells becoming more obvious with increasing passages (Figure 6A,B). In the later stages of senescence (passage 11), senescence and death were accelerated in mutant cells, while cells expressing wild type lamin A began to appear immortalized. At the same time, methyl tetrazolium salt (MTS) assays showed that cells expressing mutant lamin A exhibited slower proliferation than cells expressing wild type lamin A (Figure 6C). In addition, analysis of the senescence markers p16^INK4a^ and p21^WAF1/CIP1^ by real-time PCR revealed upregulation of p16^INK4a^ and p21^WAF1/CIP1^ in LMNA^−/−^ cells expressing lamin A with mutated Ser628 or Ser636 sites, compared with cells expressing wild type lamin A (Figure 6D). These findings were confirmed by Western blotting, which showed higher levels of p16^INK4a^ and p21^WAF1/CIP1^ proteins in LA^S628A^ and LA^S636A^ mutant cells (Figure 6E). It is worth noting that a loss of phosphorylation at these two sites affected the nuclear distribution of lamin A (Figure 6F,G). In addition, the DNA damage response was impaired, as determined by pS824–Kap-1 and γH2AX levels, which peaked ~1 h after camptothecin (CPT) treatment; this peak was dampened in LA^S628A^ and LA^S636A^ mutant cells (Figure 6H). Taken together, these findings indicate that a loss of phosphorylation at the Ser628 and Ser636 sites of lamin A leads to DNA damage and the acceleration of cellular senescence.

## 4. Discussion

The present study explored the phosphorylation of lamin A at its C-terminus and revealed important insights regarding the molecular mechanisms underlying the role of lamin A in senescence. Our study showed that the level of phosphorylation of progerin was lower than that of wild type lamin A and that phosphorylation sites within the 50 amino acids missing from progerin were involved in cellular senescence. Furthermore, we explored the impact of phosphorylation sites with high turnover rates (Ser628 and Ser636) on the phosphorylation of lamin A and confirmed that the kinases GSK3β and CK2 phosphorylated Ser628 and Ser636, respectively. We also found that phosphorylation at Ser636 influenced the phosphorylation of Ser628.

The study of protein kinases involved in the regulation of aging has been a hot topic in recent years. Our previous studies confirmed that most tissues in premature and normal aging mouse models exhibited a decreased expression of CK2 and weakened CK2 enzymatic activity is an important cause of aging [21]. This study further revealed that the loss of lamin A C-terminal phosphorylation sites regulated by CK2 directly mediated cellular senescence. Interestingly, our data showed that GSK3β cooperated with CK2 to phosphorylate lamin A. A decreased expression of GSK3β has also been identified in some aging tissues [23]; GSK3β KO mice display a metabolic phenotype, abnormal neuronal development, and accelerated aging, and die in embryogenesis or at birth [24]. GSK3β participates in multiple aging-related pathways, including mTOR, NFκB, p53, AMPK, and PGC-1α, and plays important roles in cell fate decisions, energy and nutrient metabolism, inflammation, and autophagy [23,25]. Recently, age-related increases in GSK3β expression, driving podocyte senescence and glomerular aging, seem opposite to functions of CK2 [24], demonstrating that GSK3β is a multifunctional enzyme, and its effects may vary in different tissues and protein targets. Nonetheless, our findings show that protein kinases cooperatively participate in the regulation of the aging process, potentially providing a strategy for drug targeting to rescue the phosphorylation of aging-related proteins at specific sites.

The next question we sought to answer was whether phosphorylation defects at these sites could play a role in the changes in nuclear localization, genomic instability, and subsequent cellular senescence induced by progerin. The experimental results provided preliminary confirmation that blocking the phosphorylation of Ser628 and Ser636 affected the nuclear distribution of lamin A. During cell division, phosphorylation at Ser22 and Ser392 mediates the depolymerization and dissolution of lamin A; interestingly, lower phosphorylated forms of Ser 22 also present in the nuclear interior during interphase [19]. Lamin dimers interact in a head-to-tail fashion, and N- and C-terminals of the proteins may influence each other based on space adjacent relations. Recent studies found interphase phosphorylation of lamin A depends dynamically on the cell’s microenvironment. Progerin was influenced to form spherical intranuclear lamin A droplets that accumulate protein kinases to phosphorylate lamin A at Ser 22 [26], leading to impaired lamin A structures. In addition, the lamin A C-terminal tail domain harbors various protein and DNA interacting sites, and phosphorylated lamins may act as transcriptional activators at enhancers in the nuclear interior [27]. In lamin A^−/−^ MEF cells supplemented with lamin A cell lines, during interphase, we observed that lamin A was diffusely distributed in the nucleus and exhibited punctate aggregation; however, punctate aggregation was not observed in LA^S628A^ and LA^S636A^ mutant cells. Moreover, foci in the lamin A-expressing cells were clustered around the perinuclear heterochromatin (Figure 6F,G), implying that phosphorylation of lamin A at Ser628 and Ser636 may be critical for maintaining heterochromatin structure and dynamics.

Nuclear lamins exhibit a high affinity to DNA and chromatin, which participate in various cellular processes including chromosome organization, transcriptional regulation, DNA damage response, cell cycle regulation, and mechanosensing [5]. With regard to the potential mechanisms by which phosphorylation defects lead to cellular senescence, DNA damage is known to be an important cause of premature aging as well as normal aging. The C-terminal of prelamin A post-translational maturation is involved in the DNA damage response. Aberrant accumulation of prelamin A at the early stages of the stress response reduces H2AX phosphorylation and 53BP1 recruitment/release, thus contributing to cellular senescence and accelerated organismal aging in progeroid laminopathies [28]. Our previous studies found that heterochromatic structure is closely related to the DNA damage repair pathway in cells undergoing premature aging [29]. ATM signaling is critical for the repair of DNA double-strand breaks (DSBs) in heterochromatin, acting via phosphorylation of the damage-associated heterochromatin protein KAP-1 (at Ser824), which co-localizes with the DNA damage response protein γH2AX at damage sites [30]. In our experiments, DNA damage resulted in lower levels of pS824KAP-1 and γ-H2AX in late-passage cells expressing Ser628/Ser636-mutated lamin A compared with cells expressing wild type protein. These findings suggested that a loss of lamin A phosphorylation at these two sites caused insensitivity to DNA damage, affecting signaling by the damage-associated heterochromatin protein, KAP-1. These features are similar to the abnormalities observed in prematurely aging cells, which, in turn, affect the process of DNA damage-induced chromatin remodeling [30]. *LMNA*^−/−^ mice also exhibited severe defects in the rapid phase of DSB repair, affecting the recruitment of repair factors to the site of injury. Interestingly, studies have revealed that *LMNA*^−/−^ mice do not exhibit a complete knockout of lamin A, as low levels of truncated lamin A protein (with deletion of exons 8–11) have been observed in vivo [31]. Significantly, several of the phosphorylation sites we identified were located in the same deleted region. Thus, C-terminal phosphorylation defects may inhibit the binding of lamin A to proteins involved in DNA damage repair and adversely affect the DNA repair pathway, accumulating cell senescence; in another word, lamin A phosphorylation sites are essential for this response. Despite these data, how CK2α and GSK3β precisely regulate DNA damage response and cellular senescence through these specific phosphorylation sites at lamin A and whether blocking it could induce cell apoptosis or mitotic catastrophe in MEFs remains to be clarified. Although SA-β- Gal is a classic method to detect cellular senescence, more methods are needed to detect the effect of lamin A phosphorylation on cellular senescence, such as GL13, which emerges as a powerful tool for cellular senescence assessment in vivo [32]. We will use this method to continue to investigate the effect of laminA phosphorylation on cell senescence in future work.

## 5. Conclusions

Overall, the present findings indicate that a loss of phosphorylation at the C-terminal of lamin A may lead to cellular senescence. Our results highlight the role of lamin A in intranuclear modification and communication and suggest a new mechanism to attenuate cellular senescence by activating the phosphorylation of lamin A using specific protein kinases. These findings may be beneficial for the future development of small-molecule drugs for use in anti-aging treatments.

## Data Availability

Not applicable.

## Supplementary Materials

The following supporting information can be downloaded at: https://www.mdpi.com/article/10.3390/cells12040639/s1, Figure S1: Isolation of LMNApro/pro MEFs. Figure S2: Prediction of lamin A phosphorylation sites using online tools. Figure S3: Bioinformatics analysis of phosphokinases acting at the Ser628 site. Figure S4: Detection of specificity of Ser628 phosphorylated antibody. Figure S5: Detection of specificity of Ser636 phosphorylated antibody. Figure S6: Construction and validation of LAS628A and LAS636A mutant cell lines.
