# Supplementary material for: Role of C-Terminal Phosphorylation of Lamin A in DNA Damage and Cellular Senescence
Source: Cells. 2023 Feb 16;12(4):639. doi: 10.3390/cells12040639 (PMC9954792; doi:10.3390/cells12040639)
Supplement: Supplementary file 1 [file cells-12-00639-s001.zip › cells-2058602-supplementary.pdf]

Supplementary Figures

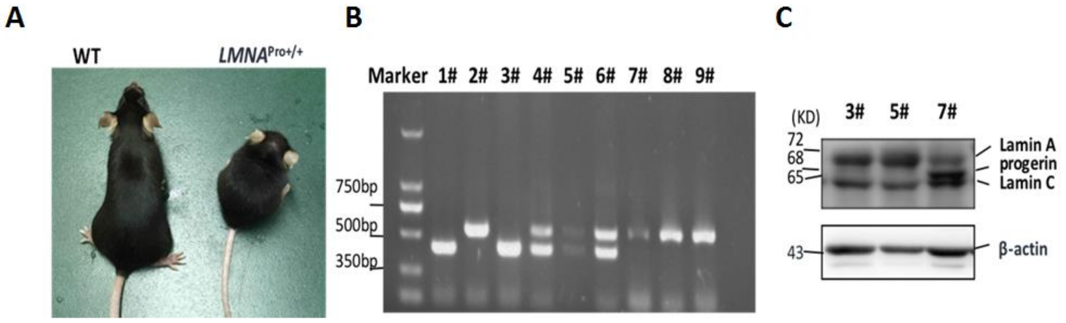

Figure S1. Isolation of LMNA$^{pro/pro}$ MEFs.

(A) Images of LMNA$^{pro/pro}$ and wild-type control mice at 4 months of age. (B) Genotype of MEF cells isolated from 13.5-day-old embryos obtained from LMNA$^{+/pro}$ intercrossed mice (C57BL background). (C) Immunoblot analysis of lamin A and progerin expression in LMNA$^{pro/pro}$ and wild-type MEFs.

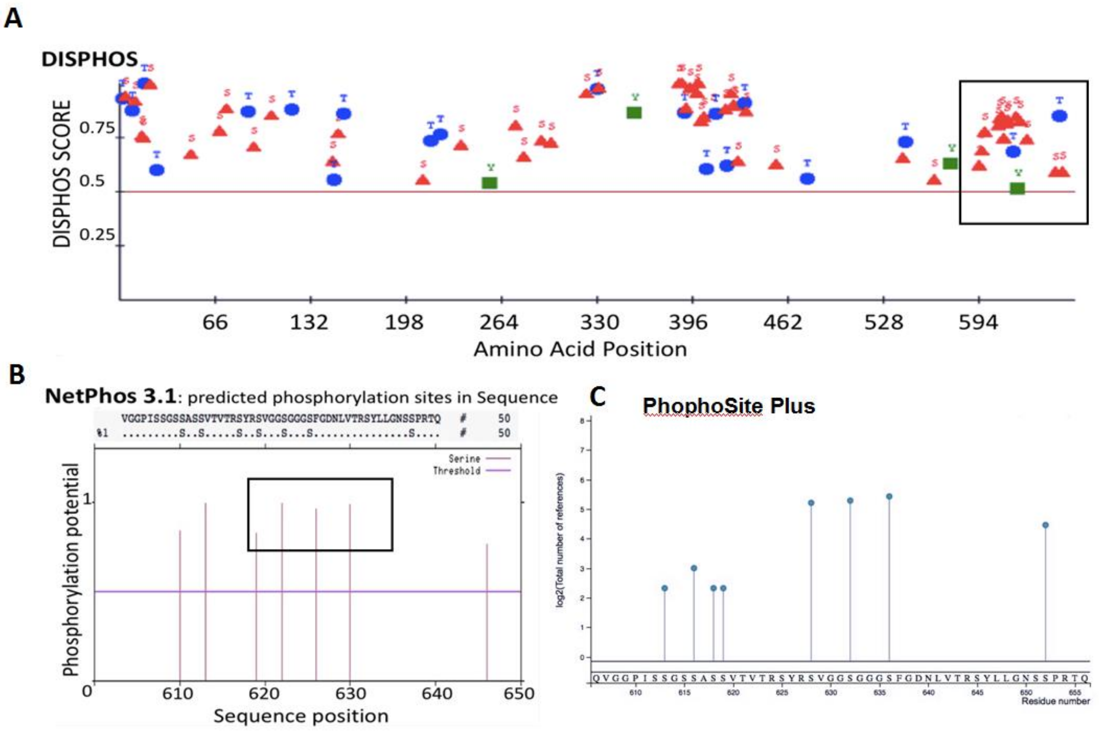

Figure S2. Prediction of lamin A phosphorylation sites using online tools.

(A) Prediction of phosphorylation sites by DISPHOS. (B) Prediction of phosphorylation sites by NetPhos 3.1. (C) Prediction of phosphorylation sites by

PhophoSite Plus.

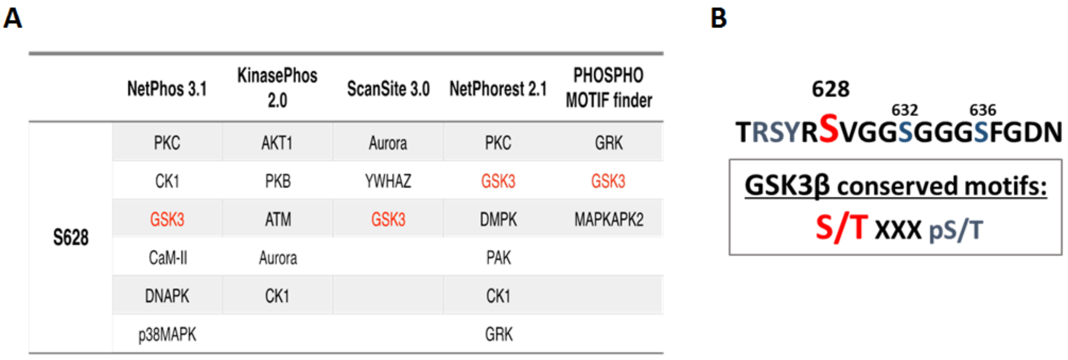

Figure S3. Bioinformatics analysis of phosphokinases acting at the Ser628 site.

(A) Predictions of phosphokinases acting at the lamin A Ser628 site. (B) The kinase recognition motif of GSK3 (pS, phosphorylated serine; S/T, target serine and threonine residues of the kinase; X, any amino acid).

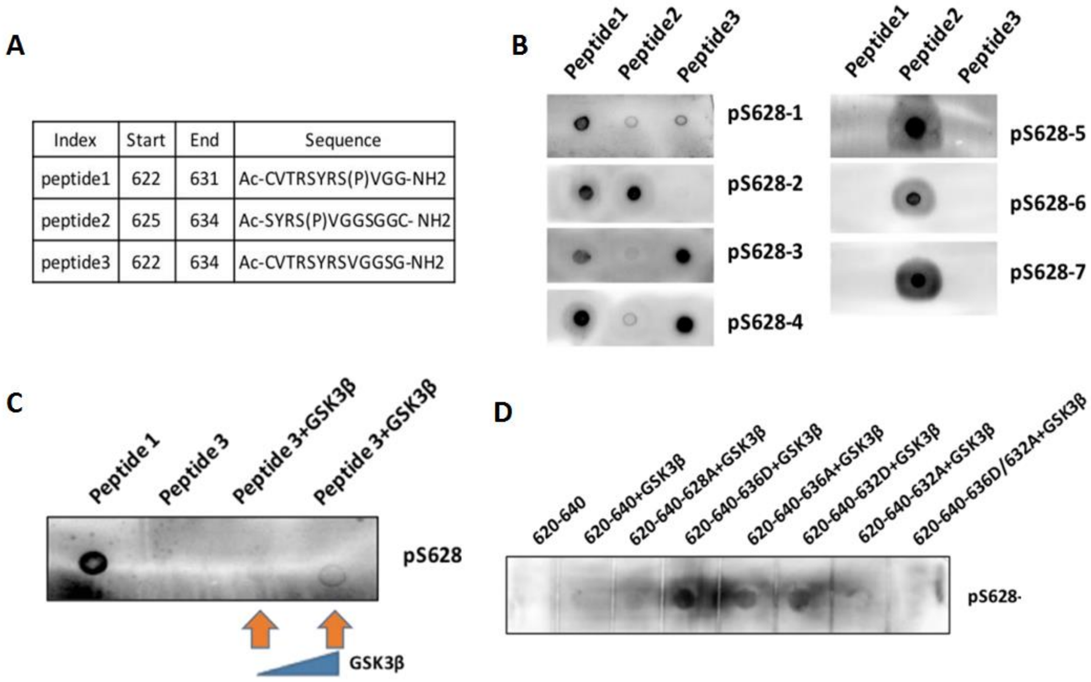

Figure S4. Detection of specificity of Ser628 phosphorylated antibody.

(A) Peptide sequences for preparation of phosphorylated Ser628-specific antibody. (B) The antibody titers of seven different ascites were detected by dot blot. (C)

Phosphorylation at Ser628 was detected by in vitro kinase assay; phosphorylation levels were detected using phosphorylated Ser628-specific antibody. (D) The effect of Ser636 on the phosphorylation of ser628 was detected by kinase assay in vitro; phosphorylation levels were detected using phosphorylated Ser628-specific antibody.

A

Index	Start	End	Sequence
peptide1	630	639	Ac-CGGSGGGS(p)FGD-NH2
peptide2	633	642	Ac-GGGS(p)FGDNLVC- NH2
peptide3	630	642	Ac-CGGSGGGSFGDNLV-NH2

B

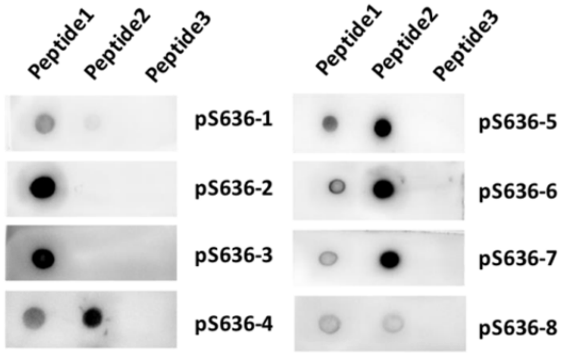

Figure S5. Detection of specificity of Ser636 phosphorylated antibody.

(A) Peptide sequences for preparation of phosphorylated Ser636-specific antibody. (B) The antibody titers of eight different ascites were detected by dot blot.

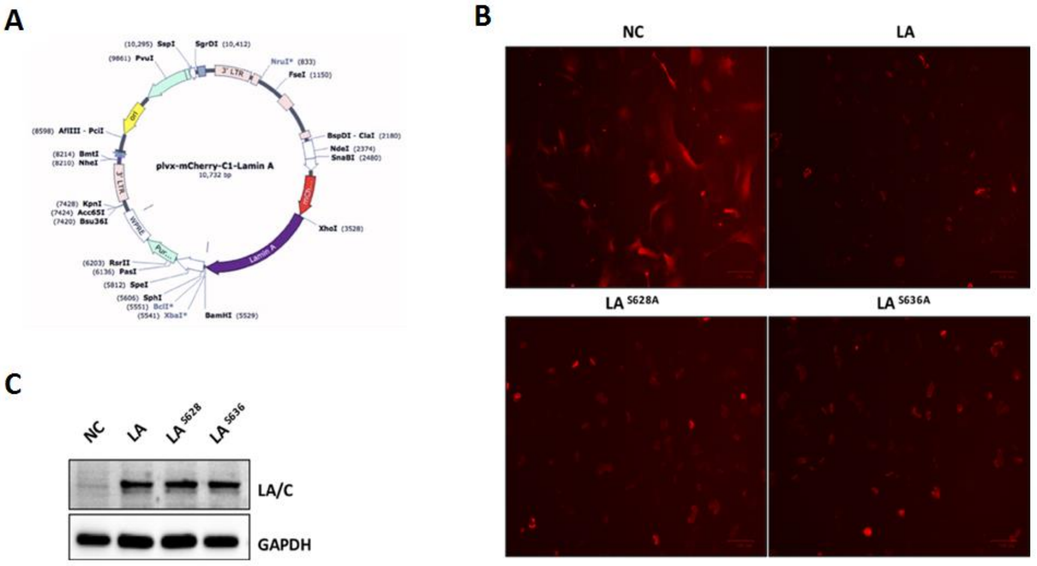

Figure S6. Construction and validation of LA^{S628A} and LA^{S636A} mutant cell lines.

(A) Map of pLVX-mCherry-C1-lamin A plasmid. (B) mCherry fluorescence of primary MEF cells infected with lentiviral constructs expressing wild-type lamin A or

S628A/S636A-mutated lamin A. NC is empty vector. (C) Immunoblots of mouse embryonic fibroblasts (MEFs) transfected with lentiviral S628A/S636A-mutated lamin A or wild-type lamin A constructs. NC is empty vector.
